# A systematic review of tests of empathy in medicine

**DOI:** 10.1186/1472-6920-7-24

**Published:** 2007-07-25

**Authors:** Joanne M Hemmerdinger, Samuel DR Stoddart, Richard J Lilford

**Affiliations:** 1 Department of Public Health and Epidemiology, University of Birmingham, Edgbaston, Birmingham, UK

## Abstract

**Background:**

Empathy is frequently cited as an important attribute in physicians and some groups have expressed a desire to measure empathy either at selection for medical school or during medical (or postgraduate) training. In order to do this, a reliable and valid test of empathy is required. The purpose of this systematic review is to determine the reliability and validity of existing tests for the assessment of medical empathy.

**Methods:**

A systematic review of research papers relating to the reliability and validity of tests of empathy in medical students and doctors. Journal databases (Medline, EMBASE, and PsycINFO) were searched for English-language articles relating to the assessment of empathy and related constructs in applicants to medical school, medical students, and doctors.

**Results:**

From 1147 citations, we identified 50 relevant papers describing 36 different instruments of empathy measurement. As some papers assessed more than one instrument, there were 59 instrument assessments. 20 of these involved only medical students, 30 involved only practising clinicians, and three involved only medical school applicants. Four assessments involved both medical students and practising clinicians, and two studies involved both medical school applicants and students.

Eight instruments demonstrated evidence of reliability, internal consistency, and validity. Of these, six were self-rated measures, one was a patient-rated measure, and one was an observer-rated measure.

**Conclusion:**

A number of empathy measures available have been psychometrically assessed for research use among medical students and practising medical doctors. No empathy measures were found with sufficient evidence of predictive validity for use as selection measures for medical school. However, measures with a sufficient evidential base to support their use as tools for investigating the role of empathy in medical training and clinical care are available.

## Background

The term 'empathy' refers to an aspect of personality that has an important role within interpersonal relationships and in facilitating competence in communication. Communication competence "has been cited consistently as a principal element or dimension"[1] of quality within the profession of medicine. Empathy is generally accepted as a desirable trait in medics and there are increasing calls to assess the level of empathy at some point during medical school, or prior to admission. Indeed empathy is a prominent attribute nominated by career counsellors in schools for people entering the medical profession[2]. Currently medical students are accepted into medical school primarily on the basis of their achieved academic grades and cognitive skills[3]. Standardised testing protocols are now very common, with most being based on cognitive abilities such as reasoning. Recently a standard test has been used by a small number of UK schools, the Medical School Admissions Test (MSAT), which includes a section explicitly seeking to measure empathy. Given the pressure to use empathy measures in the selection of medical students and the fact that some medical school selection already includes such tests, we decided to review the current literature concerning empathy measurement in medicine.

### Conceptualising empathy

Before we describe our methods and results it is necessary to conceptualise empathy in more detail. Empathy is a personality trait that enables one to identify with another's situation, thoughts, or condition by placing oneself in their situation. Empathy can be confused with sympathy. The distinction between the terms 'empathy' and 'sympathy' has been summarised thus: "empathetic physicians share their understanding, while sympathetic physicians share their emotions with their patients"[4]. That said, the precise nature of empathy is not altogether clear. Issues such as whether or how it may differ from constructs such as 'emotional competence' or 'patient centeredness' have been discussed in detail elsewhere, particularly in the field of nursing [5,6], as have questions regarding the dimensionality of empathy and it's relation to social [7] and clinical [8] function. The difficulty in finding a single agreed definition of the empathy construct has consequences for this review both in terms of structuring the review itself and in terms of defining validity; where variations exist in the definition of a construct, approaches to assessing construct, and even criterion, validity may differ markedly. Indeed, the definition of 'emotional intelligence' as the 'Ability to monitor one's own and other people's emotions...and to use emotional information to guide thinking and behaviour'[9] is sufficiently close to some definitions of empathy to warrant the inclusion of the terms 'emotional intelligence' and 'emotional quotient' in a systematic review.

To maximise the general relevance of our review, we started from the non-specific definition that empathy is an attribute related to the understanding and communication of emotions in a way that patients value. Therefore, a measurement tool for use in selection or training for empathy should measure emotional attributes that patients would value. Such attributes are likely to enhance patient satisfaction, adherence to therapy, and willingness to divulge sensitive information that may assist diagnosis. This implies that a valid tool would not measure only the ability to understand emotion, but also to do so in a way that elicits reciprocal positive emotions in the patient. Therefore, we consider a valid test as one that will predict how well the doctor will perform in the emotional area through the eyes of patients. We thus regard predictive validity, in the form of 'patient validation', as the most salient dimension of construct delivery when evaluating empathy tests in the context of selection. This is considered in more detail in the description of our data extraction methods.

Empathy may be measured from three different perspectives:

• Self-rating (first person assessment) – the assessment of empathy using standardised questionnaires completed by those being assessed.

• Patient-rating (second person assessment) – the use of questionnaires given to patients to assess the empathy they experience among their carers.

• Observer rating (third person assessment) – the use of standardised assessments by an observer to rate empathy in interactions between health personnel and patients, including the use of 'standardised' or simulated patient encounters to control for observed differences secondary to differences between patients.

Clearly, the feasibility of a particular type of test will depend, to a large extent, on the situation in which it is to be used. For example, second or third person tests are unlikely to be practical for screening many thousands of medical school applicants. However, there would be fewer logistical constraints on using such tests to help medical students or recently qualified doctors to choose a specialty or as a means of examination or continuous professional assessment. This systematic review was conducted to identify evidence about the psychometric properties of tests assessing empathy from all three perspectives, but with a particular emphasis on self-completed questionnaires, given the topicality of such tests in medical student selection.

## Methods

The search procedure for the systematic review is described in Additional file 1 [see Additional file 1].

### Search strategy

**Figure 1:**
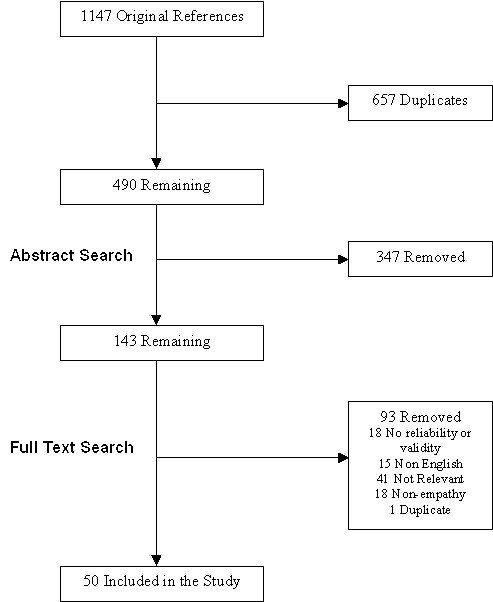
Flow diagram of paper selection process.

The initial search and abstract screen was conducted by SS in January 2005, with articles retrieved either if they were considered to be relevant on the basis of the abstract or if the abstract did not provide sufficient information on which a judgement could be based. SS and JH then screened full-text articles. A second search, in 2007, was performed in order to update the review and was supplemented with additional articles known to the reviewers of this paper. A flow diagram of the inclusion/exclusion procedure can be seen in Figure 1.

Papers were included if they met the following criteria, determined by the scope of the review:

• Groups tested were applicants to medical school, medical students, or doctors.

• Test reliability and/or validity were assessed.

• The test used measured empathy, emotional intelligence or emotional quotient. Papers were excluded, on the grounds that we did not have the resources to pursue them, if they met the following criteria:

• The paper was written in a language other than English.

• Not a published paper (e.g. theses and dissertations).

• Paper published prior to 1980.

### Data extraction

We extracted information from each paper into a spreadsheet. Data collected included:

• Bibliographic information.

• A description of the test.

• Classification of the test (1^st^, 2^nd^, or 3^rd ^person).

• Sample tested (e.g. medical students).

• Validity assessments (as described below).

• Reliability and internal consistency estimates.

*Reliability *is a measure of the tendency of a test to provide consistent results when applied under differing conditions, but where the same result should arise. Examples of this include *inter-rater reliability*, which is the degree to which different raters produce the same results when independently rating an individual, and *test-retest reliability*, which is the tendency of a test to produce the same result for the same individual on different occasions. It is worth noting that the type of reliability assessment used will depend, to some extent, on the type of test used. For example, 2^nd ^person measures are unlikely to have been assessed for inter-rater reliability as differences between patients' ratings of a clinician may arise from multiple sources (e.g. differences in the content of the consultations). This would necessitate a rather more complex statistical approach to the inter-rater reliability of this group of tests. *Internal consistency *(sometimes considered a form of reliability) is a measure of the extent to which the constituent parts of a test give consistent results (e.g. whether individual questions produce similar scores).

*Validity *refers to the quality of the mapping between the test and the quality (in this case empathy) that the test purports to measure. There are many classes of evidence that bear on validity, and we used the classification system described in Table 1. In addition we noted any evidence of first person tests being validated against patient reports of empathy or satisfaction. We refer to this measure of predictive validity as *patient validation*. The rationale for this was that selection on the basis of 'empathy' is predicated on the assumption that more empathic clinicians (as judged by the test) will provide a better patient experience. If this cannot be demonstrated, the logic of empathy tests for selection must be called into question.

**Table 1:** Classification of validity evidence

1.	**Convergent/concurrent validity **– *Convergent validity *is usually used to refer to the extent to which theoretically related tests (e.g. tests of numerical and verbal intelligence) correlate. *Concurrent validity *refers to the extent to which two (or more) tests of the same construct (e.g. two verbal intelligence tests) correlate. Given the conceptual difficulties in defining empathy precisely, and the subtly different definitions used by different groups, distinguishing between convergent and concurrent validity was difficult and unlikely to be reliable. Therefore, convergent and concurrent validity were classed together.
2.	**Divergent validity **– This is a measure of validity based on the principle that theoretically unrelated constructs should correlate poorly. For example, some groups proposed that empathy scores should not correlate with scores on tests of biomedical knowledge as they treated the two constructs as independent.
3.	**Formally assessed face validity **– This is a measure of the extent to which the test appears to assess the construct of interest. Any formal process of assessing the extent to which a relevant group (e.g. patients, clinicians) recognised the test as measuring 'empathy' was considered in this class.
4.	**Factor analysis **– Factor analysis may be treated as a means of assessing convergent and divergent validity *within *the test under consideration. It involves the statistical analysis of inter-item correlations in order to identify underlying structures within the test. This is usually in the form of subscales containing test items that correlate highly with one another but less well with other items in the test. However, factor analysis is open to accusations of 'results fixing' because of the number of statistical decisions to be made before a result is produced and may, without an agreed theoretical framework, be difficult to interpret. For this reason, factor analysis was treated as a separate validation tool.
5.	**Other validity **– This class of validation methods was used to capture validity assessments that, while of potential relevance, were unusual or not intrinsically relevant. For example, some groups, on the basis of previous findings of an empathy differential between men and women, used a difference in empathy scores between men and women as a measure of criterion-related validity.

Please note that convergent and divergent validity, together with factor analysis, give an indication of construct validity. We have avoided terms such as construct validity and criterion-related validity because readers are likely to disagree over the nature of the construct 'empathy' and the suitability of various validating criteria. Interested readers are advised to read a general introduction to validity, such as that provided by David Clark-Carter [64].

In extracting data from the literature, we observed:

• Whether empathy was treated as a continuous, ordinal, or categorical variable.

• The time interval between initial empathy testing and subsequent tests for reliability and validity.

• Any subgroup analyses, such as analysis by ethnic group.

## Results

A summary of the article screening process is presented in figure 1, and information regarding all 36 tests is presented in an additional file [see Additional file 2].

Of the 36 identified tests, 14 were first person assessments, 5 were second person assessments, and 17 were third person assessments. 59 assessments of empathy measures were performed. The study groups for these were:

• Medical school students only = 20

• Medical school applicants only = 3

• Practising clinicians only = 30

• Medical school students and practising clinicians = 4

• Medical school applicants and students = 2.

We selected for further analysis empathy tests for which the basic psychometric evidence of reliability (inter-rater or test-retest) and internal consistency was present and for which at least one of the validity assessments described in Table 1 had been carried out. This evidence could come from multiple papers and was based on simply checking which tests had the requisite ticks in Additional file 2 [see Additional file 2] and collating the information from the data extraction forms. The result was a selection of eight tests with the greatest evidential base [see Additional file 3]. The majority of these measures (6 out of 8) concerned first-person assessment questionnaires of the type that might be useful for screening large numbers of applications to study medicine, while one concerned empathy from the patient perspective, and one involved a third-person assessment of empathy.

### First person measures

The assessment of test-retest reliability for a first-person test involves measuring changes in test score over time. If the interval between tests is short, then the results may be affected by memory of previous answers. However, individual changes in score over longer time periods will consist of both random changes (due to poor reliability inherent in the test) and non-random changes (due to learning, maturation, training, or other time-related factors). The studies reported here included two approaches to assessing test-retest reliability:

• Standard correlation methods, such as Pearson's r, were used in three studies of first person measures. Such methods do not provide information on systematic differences over time (e.g. due to learning) but measure linear association between pairs of values; the lower the correlation, the greater the change in rank order on retesting.

• Four of the first person tests were investigated for differences over time using paired tests. In two studies, Wilcoxon's signed rank test was used, while another study appears to have been based on a paired t-test [10] and the fourth involved a repeated measures ANOVA [11].

The interval between test and re-test for correlations was 17 days, 4 months, and 12 months across the three tests (MCRS, JSPE, and ET) respectively. Tests used for selection purposes should have high correlation; even a correlation of r = 0.84 still implies that 29% of score variation is random.

Paired tests were conducted on four of the first person measures: the JSPE, the ECRS, the DIRI, and the BEES. Statistically significant changes were not observed for the ECRS over 6 months, although the sample size was very small (n = 16), limiting power. Statistically significant changes over time were observed for the JSPE, the DIRI and the BEES, with JSPE scores declining over a 12-month period, DIRI scores declining over 3 years and BEES scores increasing over 6 months.

First person measures generally had adequate internal consistency, although the Empathy Test was an exception with Cronbach's alpha statistics between 0.18 and 0.42 [12].

Validity assessments of first-person measures were primarily concerned with assessing the relationship between measured empathy and various aspects of the consultation or clinical knowledge. None of the first person measures were validated by directly comparing measured empathy with empathy as judged by patients, although the JSPE was subjected to a test of predictive validity through correlating empathy scores with later ratings of empathy from directors during residencies [13]. Correlations between first-person measures of empathy were, where available, not large [see Additional file 3] [14,15].

### Second and third person measures

The only second person measure with evidence of reliability, internal consistency and validity was the CARE, which showed excellent internal consistency, and was relatively comprehensively validated in terms of both content/face validity and convergent validity [16]. In addition, there was some evidence that measured empathy was related to other aspects of the patient experience [17-19]. Test-retest correlations over 3 months were not very large (rho = 0.572), although staff changes may influence second person ratings at subsequent visits and so result in an underestimate of reliability. An interesting finding was that the variance (i.e. spread) of patient ratings appeared to be dependent upon the score given, such that patients tended to agree on high empathy scores more than on low empathy scores [18].

The Four Habits Coding Scheme (FHCS) was also relatively comprehensively investigated in terms of convergent validity and proved reasonably reliable and internally consistent [20]. The FHCS was correlated with patient evaluations of care, but correlations were very weak (-0.17 < r < 0.03) and not statistically significant [20].

## Discussion

From a systematic search of the literature pertaining to empathy assessment in medicine, we identified 50 papers reporting on 36 different tests of empathy. Eight of these tests had evidence concerning reliability (including internal consistency) and validity. The first person tests do not appear to be very reliable over periods of 4 to 12 months. Not only do the mean results change over time, but they are poorly correlated, so the rank order of those being tested may not remain constant. For example, the Empathy Test showed test-retest reliability of 0.37 over 12-month periods,[12] suggesting that it is not measuring a stable personality construct, or is doing so poorly.

One reassuring finding was that there is a second person measure, the CARE measure, that has been subjected to sufficient psychometric evaluation to be considered a useful measure of empathy from the patient's perspective. This is particularly useful as it may aid in the development of first person measures of empathy and, together with third person measures of empathy, enrich our understanding of the role empathy plays in the care process.

The data do not allow us to compare reliability or validity by sub-groups (e.g. personality type, ethnic group). We acknowledge that limiting our search to English language publications precludes the possibility of examining any differences in the way empathy tests may play out across very different social contexts. It is also important to note that any systematic review may miss important literature. We have not been able to conduct extensive grey or unpublished literature searches and we are aware that a review of this type will tend to be biased against measures that are still undergoing longitudinal evaluation (for example, the NACE [21]).

We also observe that, in all cases, the statistical tests we found in the literature treated empathy as a continuous variable; the results were not, for example, categorised into high and low (good and bad). However, empathy used as a criterion for selection could be dichotomised. That is to say, empathy could be used as a 'gating' criterion to identify a (small) number of people falling below a certain threshold, rather than as a 'weighting' criterion to be combined with other information in the assessment of all applicants. Such a situation might be appropriate where a measure is poor at discriminating between individuals within the normal range but can reliably detect sizeable impairments in social functioning. The justification of using gating to screen applicants is, of course, dependent on other information, such as the base-rate of poor empathy within the population tested, that is independent of the test itself.

Increasingly, characteristics such as empathy are being explicitly assessed during the selection of medical students. While it may be admirable that standardised approaches are replacing informal assessments of these same characteristics, the evidence available does not suggest that any existing empathy measures are sufficiently reliable and valid for pre-training selection. This is in addition to questions as to potential costs of selecting for empathy itself, such as the question of whether more empathy is always better or, indeed, whether a display of empathy is accompanied by a genuine concern (i.e. whether emotional expression is honest).

In our opinion, demonstrating predictive validity would be a necessary, but not a sufficient, criterion for use of an empathy test for selection purposes. This is because the psychometric properties of a test may change according to the context in which it is used[22]. That is to say, a test for empathy may behave differently when the results can affect a person's life chances as opposed to when the test is used for other (less critical) purposes. In particular, biased responding on personality tests can occur [23-25] even when measures are taken to reduce faking[26] and it is very likely that medical applicants are capable of 'cheating the test'. A reliable and valid empathy tool, if one can be produced, would be useful in research, training, and self-assessment, but it would need to be highly resistant to faking if used to select medical students. It may be the case that there exists a proportion of people who are unable even to fake the test and that this group would manifest poor doctor-patient relationships later in life. It would be hard to test this hypothesis directly, but a necessary first step would be to see if there is a group of people who perform poorly both on testing (when not used for selection) and then, later, in patients' eyes. We have embarked on such a study here at the Birmingham Medical School.

## Conclusion

• Empathy is considered to be an important quality in doctors and there have been moves to include measures of empathy in the selection process for medical students.

• Despite this, we found no systematic reviews of the use of empathy tests on doctors or potential doctors.

• There is insufficient evidence to support the use of empathy tests in the selection of students for medical courses.

## Competing interests

The author(s) declare that they have no competing interests.

## Authors' contributions

JH collated data, extracted data, and drafted the paper. SS devised the search strategy, participated in data collation/extraction, and redrafted the final manuscript. RL proposed the study and conceptual approach, directed the research, and commented on and corrected both draft and final versions of the paper.

## Pre-publication history

The pre-publication history for this paper can be accessed here:



## Supplementary Material

Additional file 1 **Search strategy**. A summary of the search strategy used for the systematic review. Click here for file

Additional file 2 **Empathy tests identified**. A summary of the tests identified by the review, illustrating the presence or absence of evidence concerning reliability and validity. Click here for file

Additional file 3 **Measures with evidence of reliability, validity, and internal consistency**. A summary of the findings regarding measures with evidence of reliability, validity, and internal consistency. Click here for file

## References

[B1] Redmond MV (1985). The relationship between perceived communication competence and perceived empathy. Communication Monographs.

[B2] Marley J, Carmen I (1999). Selecting medical students: a case report of the need for change. Medical Education.

[B3] McManus IC, Powis DA, Wakeford R, Ferguson E, James D, Richards P (2005). Intellectual aptitude tests and A levels for selecting UK school leaver entrants for medical school. British Medical Journal.

[B4] Hojat M, Gonnella JS, Nasca TJ, Mangione S, Vergare M, Magee M (2002). Physician empathy: definition, components, measurement, and relationship to gender and specialty. American Journal of Psychiatry.

[B5] Evans GW, Wilt DL, Alligood MR, O'Neil M (1998). Empathy: a study of two types. Issues in Mental Health Nursing.

[B6] Kunyk D, Olson JK (2001). Clarification of conceptualizations of empathy. Journal of Advanced Nursing.

[B7] Cliffordson C (2002). The hierarchical structure of empathy: Dimensional organization and relations to social functioning. Scandinavian Journal of Psychology.

[B8] Suchman AL, Markakis K, Beckman HB, Frankel R (1997). A model of empathic communication in the medical interview. Journal of the American Medical Association.

[B9] Colman AM (2001). Dictionary of Psychology.

[B10] Hojat M, Mangione S, Nasca TJ, Rattner S, Erdmann JB, Gonnella JS, Magee M (2004). An empirical study of decline in empathy in medical school. Medical Education.

[B11] Bellini LM, Shea JA (2005). Mood change and empathy decline persist during three years of internal medicine training. Academic Medicine.

[B12] Feletti GI, Sanson-Fisher RW, Vidler M (1985). Evaluating a new approach to selecting medical students. Medical Education.

[B13] Hojat M, Mangione S, Nasca TJ, Gonnella JS, Magee M (2005). Empathy scores in medical school and ratings of empathic behavior in residency training 3 years later. The Journal of Social Psychology.

[B14] Hojat M, Mangione S, Kane GC, Gonnella JS (2005). Relationships between scores of the Jefferson Scale of Physician Empathy (JSPE) and the Interpersonal Reactivity Index (IRI). Medical Teacher.

[B15] Shapiro J, Morrison E, Boker J (2004). Teaching empathy to first year medical students: evaluation of an elective literature and medicine course. Education for Health.

[B16] Mercer SW, Maxwell M, Heaney D, Watt GC (2004). The consultation and relational empathy (CARE) measure: development and preliminary validation and reliability of an empathy-based consultation process measure. Family Practice.

[B17] Bikker AP, Mercer SW, Reilly D (2005). A pilot prospective study on the consultation and relational empathy, patient enablement, and health changes over 12 months in patients going to the Glasgow Homeopathic Hospital. J Altern Complement Med.

[B18] Mercer SW, McConnachie A, Maxwell M, Heaney D, Watt GCM (2005). Relevance and practical use of the Consultation and Relational Empathy (CARE) measure in general practice. Family Practice.

[B19] Mercer SW, Howie JGR (2006). CQI-2 – a new measure of holistic interpersonal care in primary care consultations. British Journal of General Practice.

[B20] Krupat E, Frankel R, Stein T, Irish J (2006). The Four Habits Coding Scheme: Validation of an instrument to assess clinicians' communication behavior. Patient Educ Couns.

[B21] Powis D, Bore M, Munro D, Lumsden MA (2005). Development of the personal qualities assessment as a tool for selecting medical students. Journal of Adult and Continuing Education.

[B22] Douglas SP, Nijssen EJ (2003). On the use of "borrowed" scales in cross-national research: a cautionary note. International Marketing Review.

[B23] Ellingson JE, Sackett PR, Hough LM (1999). Social desirability corrections in personality measurement: Issues of applicant comparison and construct validity. Journal of Applied Psychology.

[B24] Alliger GM, Dwight SA (2000). A meta-analytic investigation of the susceptibility of integrity tests to faking and coaching. Educational and Psychological Measurement.

[B25] Viswesvaran C, Ones DS (1999). Meta-analyses of fakability estimates: Implications for personality measurement. Educational and Psychological Measurement.

[B26] Heggestad ED, Morrison M, Reeve CL (2006). Forced-choice assessments of personality for selection: Evaluating issues of normative assessment and faking resistance. Journal of Applied Psychology.

[B27] Christison GW, Haviland MG, Riggs ML (2002). The medical condition regard scale: measuring reactions to diagnoses. Academic Medicine.

[B28] Buddeberg-Fischer B, Klaghofer R, Abel T, Buddeberg C (2003). The influence of gender and personality traits on the career planning of Swiss medical students. Swiss Medical Weekly.

[B29] Hojat M, Gonnella JS, Mangione S, Nasca TJ, Veloski JJ, Erdmann JB, Callahan CA, Magee M (2002). Empathy in medical students as related to academic performance, clinical competence and gender. Medical Education.

[B30] Hojat M, Gonnella JS, Nasca TJ, Mangione S, Veloksi JJ, Magee M (2002). The Jefferson Scale of Physician Empathy: further psychometric data and differences by gender and specialty at item level. Academic Medicine.

[B31] Fields SK, Hojat M, Gonnella JS, Mangione S, Kane G, Magee M (2004). Comparisons of nurses and physicians on an operational measure of empathy. Evaluation & the Health Professions.

[B32] Hojat M, Mangione S, Nasca TJ, Cohen MJM, Gonnella JS, Erdmann JB, Veloski J, Magee M (2001). The Jefferson Scale of Physician Empathy: Development and preliminary psychometric data. Educational and Psychological Measurement.

[B33] Coman GJ, Evans BJ, Stanley RO (1988). Scores on the Interpersonal Reactivity Index: A sample of Australian medical students. Psychological Reports.

[B34] Morton KR, Worthley JS, Nitch SR, Lamberton HH, Loo LK, Testerman JK (2000). Integration of cognition and emotion: A postformal operations model of physician-patient interaction. Journal of Adult Development.

[B35] Elam C, Stratton TD, Andrykowski MA (2001). Measuring the emotional intelligence of medical school matriculants. Academic Medicine.

[B36] West CP, Huschka MM, Novotny PJ, Sloan JA, Kolars JC, Habermann TM, Shanafelt TD (2006). Association of perceived medical errors with resident distress and empathy: A prospective longitudinal study. Journal of the American Medical Association.

[B37] Shanafelt TD, West C, Zhao X, Novotny P, Kolars J, Habermann T, Sloan J (2005). Relationship between increased personal well-being and enhanced empathy among internal medicine residents. Journal of General Internal Medicine.

[B38] McManus IC, Livingston G, Katona C (2006). The attractions of medicine: the generic motivations of medical school applicants in relation to demography, personality and achievement. BMC Medical Education.

[B39] Holm U (1996). The Affect Reading Scale: A method of measuring prerequisites for empathy. Scandinavian Journal of Educational Research.

[B40] Shapiro SL, Schwartz GE, Bonner G (1998). Effects of mindfulness-based stress reduction on medical and premedical students. Journal of Behavioral Medicine.

[B41] Torrubia R, Tobena A (1984). A scale for the assessment of "susceptibility to punishment" as a measure of anxiety: Preliminary results. Personality and Individual Differences.

[B42] Zeldow PB, Daugherty SR (1987). The stability and attitudinal correlates of warmth and caring in medical students. Medical Education.

[B43] Varkey P, Chutka DS, Lesnick TG (2006). The aging game: Improving medical students' attitudes toward caring for the elderly. Journal of the American Medical Directors Association.

[B44] Munro D, Bore M, Powis D (2005). Personality factors in professional ethical behaviour: Studies of empathy and narcissism. Australian Journal of Psychology.

[B45] Dawson C, Schirmer M, Beck L (1984). A patient self-disclosure instrument. Research in Nursing & Health.

[B46] Larsson G, Larsson BW (2002). Development of a short form of the Quality from the Patient's Perspective (QPP) questionnaire. Journal of Clinical Nursing.

[B47] Mercer SW (2005). Practitioner empathy, patient enablement and health outcomes of patients attending the Glasgow Homeopathic Hospital: a retrospective and prospective comparison. Wiener Medizinische Wochenschrift.

[B48] Roter DL, Larson S, Shinitzky H, Chernoff R, Serwint JR, Adamo G, Wissow L (2004). Use of an innovative video feedback technique to enhance communication skills training. Medical Education.

[B49] Hart CN, Drotar D, Gori A, Lewin L (2006). Enhancing parent-provider communication in ambulatory pediatric practice. Patient Educ Couns.

[B50] Hodges B, McIlroy JH (2003). Analytic global OSCE ratings are sensitive to level of training. Medical Education.

[B51] Silber CG, Nasca TJ, Paskin DL, Eiger G, Robeson M, Veloski JJ (2004). Do global rating forms enable program directors to assess the ACGME competencies?. Academic Medicine.

[B52] Vernooij-Dassen MJ, Ram PM, Brenninkmeijer WJ, Franssen LJ, Bottema BJ, van der Vleuten CP, Grol RP (2000). Quality assessment in general practice trainers. Medical Education.

[B53] Carrothers RM, Gregory SW, Gallagher TJ (2000). Measuring emotional intelligence of medical school applicants. Academic Medicine.

[B54] Bylund CL, Makoul G (2002). Empathic communication and gender in the physician-patient encounter. Patient Educ Couns.

[B55] Winefield HR, Chur-Hansen A (2000). Evaluating the outcome of communication skill teaching for entry-level medical students: does knowledge of empathy increase?. Medical Education.

[B56] Jenkins V, Fallowfield L (2002). Can communication skills training alter physicians' beliefs and behavior in clinics?. Journal of Clinical Oncology.

[B57] Fallowfield L, Jenkins V, Farewell V, Saul J, Duffy A, Eves R (2002). Efficacy of a Cancer Research UK communication skills training model for oncologists: a randomised controlled trial. Lancet.

[B58] Gillotti C, Thompson T, McNeilis K (2002). Communicative competence in the delivery of bad news. Soc Sci Med.

[B59] Schnabl GK, Hassard TH, Kopelow ML (1991). The assessment of interpersonal skills using standardized patients. Acad Med.

[B60] van Zanten M, Boulet JR, Norcini JJ, McKinley D (2005). Using a standardised patient assessment to measure professional attributes. Medical Education.

[B61] Wolf FM, Woolliscroft JO, Calhoun JG, Boxer GJ (1987). A controlled experiment in teaching students to respond to patients'emotional concerns. Journal of Medical Education.

[B62] Ring A, Dowrick CF, Humphris GM, Davies J, Salmon P (2005). The somatising effect of clinical consultation: What patients and doctors say and do not say when patients present medically unexplained physical symptoms. Soc Sci Med.

[B63] Shields CG, Epstein RM, Franks P, Fiscella K, Duberstein P, McDaniel SH, Meldrum S (2005). Emotion language in primary care encounters: reliability and validity of an emotion word count coding scheme. Patient Educ Couns.

[B64] Clark-Carter D (1997). Doing Quantitative Psychological Research: From Design to Report.

