# Supplementary material for: A systematic review of tests of empathy in medicine
Source: BMC Med Educ. 2007 Jul 25;7:24. doi: 10.1186/1472-6920-7-24 (PMC1988794; doi:10.1186/1472-6920-7-24)
Supplement: Additional file 1 — Search strategy. A summary of the search strategy used for the systematic review. [file 1472-6920-7-24-S1.pdf]

# Appendix 1. Search Procedure for the Systematic Review

## Databases used for Initial Search

| | |
|---|---|
| Ovid MEDLINE (R) | In-Process and other Non-Indexed Citations (January 17th 2005) |
| Ovid MEDLINE (R) | (1966 to November week 3 2004) |
| EMBASE | (1980 to 2005 week 03) |
| PsycINFO | (1872 to January week 1 2005) |

## Databases used for Updated Search

| | |
|---|---|
| Ovid MEDLINE (R) | In-Process and other Non-Indexed Citations (January 31st 2007) |
| Ovid MEDLINE (R) | (1950 to January week 4 2007) |
| EMBASE | (1980 to 2007 week 04) |
| PsycINFO | (1806 to January week 5 2007) |

The search string used is given in Table 1:

**Table 1 - Search String for Identifying Articles**

1. Empathy.mp.
2. Emotional Intelligence.mp.
3. Emotional quotient.mp
4. Validation Studies.pt.
5. Reproducibility of results.sh.
6. Valid$.mp.
7. Follow?up.mp.
8. Longitudinal.mp.
9. Prospective.mp.
10. Intervention.mp.
11. Stability.mp.
12. 1 OR 2 OR 3
13. 4 OR 5 OR 6 OR 7 OR 8 OR 9 OR 10 OR 11
14. 12 AND 13
15. Professional-patient relations.sh.
16. Physician-patient relations.sh.
17. Communication.mp.
18. medic$.mp.
19. 15 OR 16 OR 17 OR 18
20. 14 AND 19
