# Supplementary material for: A systematic review of tests of empathy in medicine
Source: BMC Med Educ. 2007 Jul 25;7:24. doi: 10.1186/1472-6920-7-24 (PMC1988794; doi:10.1186/1472-6920-7-24)
Supplement: Additional file 3 — Measures with evidence of reliability, validity, and internal consistency. A summary of the findings regarding measures with evidence of reliability, validity, and internal consistency. [file 1472-6920-7-24-S3.pdf]

Additional file 3 - Empathy tests with evidence concerning reliability, internal consistency, and validity

Test	Papers	Type of Assessment	Reliability	Internal Consistency	Validity	Notes
Medical Condition Regard Scale (MCRS)	[1]	1st Person	Test-retest **correlation** over 17 days (r=0.84, n=93)	Cronbach's alpha=0.87	Factor analysis used in test development – 11-item, single factor solution found.<br>Medical students asked to rate attitudes towards various medical conditions thought to elicit varying levels of empathy. Second validity assessment investigated changes following a psychiatry clerkship.	This test assesses attitudes towards medical conditions. This may be indicative of empathic understanding, but is not specifically an empathy test.
Jefferson Scale of Physician Empathy (JSPE)	[2-9]	1st Person	Test-retest **correlation** over 3-4months in physicians (r=0.65, n=71)[5]<br><br>Test-retest **change** over 12 months in medical students (Cohen's *d*=0.29, n=125, p<0.05)[2].	Item-total score correlations from 0.30-0.60[7]<br>Cronbach's alpha=<br>0.89[4]<br>0.81[5]<br>0.87[3]<br>0.89[3]	Association between empathy scores and assessed competence in students but no significant association with scientific knowledge[6].<br>Student empathy scores associated with later ratings of empathy by directors during residency[8].<br>Statistically significant differences in scores between specialties and, possibly, between genders[5,7].<br>Factor structure consistent with expectation[5].<br>Extensive convergent and divergent validity assessments during test development. Formal investigation of appropriate components, with piloting for face validity, and factor analysis[3].<br>Correlations with Davis' Interpersonal Reactivity Index (r=0.45 for total scales)[9].	The most heavily researched test and specifically designed from scratch for the assessment of physician empathy.
Davis' Interpersonal Reactivity Index (DIRI)	[9-16]	1st Person	Repeated 4 times over 3 years. Statistically significant **change** over 3 years of residency (effect size = -0.51, p = 0.05)[15]	Subscales Cronbach's alphas:<br>Perspective Taking = 0.77<br>Empathic Concern = 0.72<br>Personal Distress = 0.77	Samples at varying stages of medical education compared to normative data on empathy scores[10].<br>Principal components analysis used to develop a model relating empathy, tolerance for ambiguity, and clinical performance[11].<br>Correlation between DIRI and Trait Meta-Mood Scale (r=0.44)[12].<br>May be associated, weakly, with reported self-perceived	Validity assessments were largely incidental to theory/model development.

Test	Papers	Type of Assessment	Reliability	Internal Consistency	Validity	Notes
				[12]	medical errors[13].<br>Statistically significant association between well-being (high vs low) and cognitive empathy[14].<br>Factor analytic and regression models used to assess role of empathy (assessed using a revised DIRI) in choice of medicine as a subject[16].<br>Correlations between DIRI and Jefferson Scale of Physician Empathy (r=0.45 for total scales)[9].	
Empathy Test (ET)	[17]	1st Person	Test-retest **correlation** over 12 month periods for 4 cohorts: weighted mean r=0.37, total n=201 (range: 0.22-0.65)	Cronbach's alpha between 0.18 and 0.42.	Empathy scores did not correlate well with scores on other admissions measures, including measures of cognitive ability. A factor analysis is reported as supporting this, but this is not easy to interpret.	The ET was used as part of a larger screening and selection process for medical students.
Empathy Construct Rating Scale (ECRS)	[18,19]	1st Person	Test-retest **change** over 6 months (Wilcoxon Z=-1.10, n=16, p=0.27)[18]	Cronbach's alpha=0.84[18] (Shortened version=0.89)[19]	Scores correlated with BEES test (see below) at two points (r=0.14 and 0.48). Neither statistically significant, but sample size was small.	
Balanced Emotional Empathy Scale (BEES)	[18]	1st Person	Test-retest **change** over 6 months (Wilcoxon Z=-2.54, n=16, p<0.01)	Cronbach's alpha=0.81	Correlated with ECRS scores as described above.	
Consultation and Relational Empathy (CARE)	[20-23]	2nd Person	Test-retest **correlation** over 3 months (Spearman's rho=0.572, p<0.0001)[21]<br>Variance across raters of a single doctor variable dependent upon score [22]	Cronbach's alpha=0.93[20]	Correlates with Barret-Lennard Relationship Inventory (BLRI) empathy subscale (r=0.84) and Reynolds' Empathy Scale (RES) (r=0.85). Also, face/content validity assessed by patients, GPs, and experts[20].<br>Scores correlate positively with measures of patient enablement, and some association with patient outcome of homeopathic treatment[21].<br>Correlates with patient enablement, continuity of care, and consultation length, as well as patient and doctor opinions [23].<br>Judged as relevant by patients and doctors and distribution of scores is practical for use [22].	CARE draws heavily on nursing research and has been used in settings where empathy is explicitly treated as therapeutic [24].

Test	Papers	Type of Assessment	Reliability	Internal Consistency	Validity	Notes
Four Habits Coding Scheme (FHCS)	[25]	3rd Person	Inter-rater reliability = 0.72	Habit 3 (Demonstrate Empathy) Cronbach's Alpha = 0.81	Correlations with Roter's Interaction Analysis System (RIAS) and patient post-visit evaluations, as well as measures of non-verbal behaviour.	Correlations with patient evaluations are very poor.

Reference List

1. Christison GW, Haviland MG, Riggs ML: **The medical condition regard scale: measuring reactions to diagnoses.** *Academic Medicine* 2002, **77:** 257-262.

2. Hojat M, Mangione S, Nasca TJ, Rattner S, Erdmann JB, Gonnella JS *et al.*: **An empirical study of decline in empathy in medical school.** *Medical Education* 2004, **38:** 934-941.

3. Hojat M, Mangione S, Nasca TJ, Cohen MJM, Gonnella JS, Erdmann JB *et al.*: **The Jefferson Scale of Physician Empathy: Development and preliminary psychometric data.** *Educational and Psychological Measurement* 2001, **61:** 349-365.

4. Fields SK, Hojat M, Gonnella JS, Mangione S, Kane G, Magee M: **Comparisons of nurses and physicians on an operational measure of empathy.** *Evaluation & the Health Professions* 2004, **27:** 80-94.

5. Hojat M, Gonnella JS, Nasca TJ, Mangione S, Vergare M, Magee M: **Physician empathy: definition, components, measurement, and relationship to gender and specialty.** *American Journal of Psychiatry* 2002, **159:** 1563-1569.

6. Hojat M, Gonnella JS, Mangione S, Nasca TJ, Veloski JJ, Erdmann JB *et al.*: **Empathy in medical students as related to academic performance, clinical competence and gender.** *Medical Education* 2002, **36:** 522-527.

7. Hojat M, Gonnella JS, Nasca TJ, Mangione S, Veloksi JJ, Magee M: **The Jefferson Scale of Physician Empathy: further psychometric data and differences by gender and specialty at item level.** *Academic Medicine* 2002, **77:** S58-S60.

8. Hojat M, Mangione S, Nasca TJ, Gonnella JS, Magee M: **Empathy scores in medical school and ratings of empathic behavior in residency training 3 years later.** *The Journal of Social Psychology* 2005, **145:** 663-672.

9. Hojat M, Mangione S, Kane GC, Gonnella JS: **Relationships between scores of the Jefferson Scale of Physician Empathy (JSPE) and the Interpersonal Reactivity Index (IRI).** *Medical Teacher* 2005, **27:** 625-628.

10. Coman GJ, Evans BJ, Stanley RO: **Scores on the Interpersonal Reactivity Index: A sample of Australian medical students.** *Psychological Reports* 1988, **62:** 943-945.

11. Morton KR, Worthley JS, Nitch SR, Lamberton HH, Loo LK, Testerman JK: **Integration of cognition and emotion: A postformal operations model of physician-patient interaction.** *Journal of Adult Development* 2000, **7:** 151-160.

12. Elam C, Stratton TD, Andrykowski MA: **Measuring the emotional intelligence of medical school matriculants.** *Academic Medicine* 2001, **76:** 507-508.

13. West CP, Huschka MM, Novotny PJ, Sloan JA, Kolars JC, Habermann TM *et al*.: **Association of perceived medical errors with resident distress and empathy: A prospective longitudinal study.** *Journal of the American Medical Association* 2006, **296:** 1071-1078.

14. Shanafelt TD, West C, Zhao X, Novotny P, Kolars J, Habermann T *et al*.: **Relationship between increased personal well-being and enhanced empathy among internal medicine residents.** *Journal of General Internal Medicine* 2005, **20:** 559-564.

15. Bellini LM, Shea JA: **Mood change and empathy decline persist during three years of internal medicine training.** *Academic Medicine* 2005, **80:** 164-167.

16. McManus IC, Livingston G, Katona C: **The attractions of medicine: the generic motivations of medical school applicants in relation to demography, personality and achievement.** *BMC Medical Education* 2006, **6**.

17. Feletti GI, Sanson-Fisher RW, Vidler M: **Evaluating a new approach to selecting medical students.** *Medical Education* 1985, **19:** 276-284.

18. Shapiro J, Morrison E, Boker J: **Teaching empathy to first year medical students: evaluation of an elective literature and medicine course.** *Education for Health* 2004, **17:** 73-84.

19. Shapiro SL, Schwartz GE, Bonner G: **Effects of mindfulness-based stress reduction on medical and premedical students.** *Journal of Behavioral Medicine* 1998, **21:** 581-599.

20. Mercer SW, Maxwell M, Heaney D, Watt GC: **The consultation and relational empathy (CARE) measure: development and preliminary validation and reliability of an empathy-based consultation process measure.** *Family Practice* 2004, **21:** 699-705.

21. Bikker AP, Mercer SW, Reilly D: **A pilot prospective study on the consultation and relational empathy, patient enablement, and health changes over 12 months in patients going to the Glasgow Homeopathic Hospital.** *The Journal of Alternative and Complementary Medicine* 2005, **11:** 591-600.

22. Mercer SW, McConnachie A, Maxwell M, Heaney D, Watt GCM: **Relevance and practical use of the Consultation and Relational Empathy (CARE) measure in general practice.** *Family Practice* 2005, **22:** 328-334.

23. Mercer SW, Howie JGR: **CQI-2 - a new measure of holistic interpersonal care in primary care consultations.** *British Journal of General Practice* 2006, **56:** 262-268.

24. Price S, Mercer SW, McPherson H: **Practitioner empathy, patient enablement, and health outcomes: a prospective study of acupuncture patients.** *Patient Education & Counseling* 2006, **63:** 239-245.

25. Krupat E, Frankel R, Stein T, Irish J: **The Four Habits Coding Scheme: Validation of an instrument to assess clinicians' communication behavior.** *Patient Education & Counseling* 2006, **62:** 38-45.
